# Bisphosphonate‐Associated Atypical Femoral Fractures: A Description of Surgical Techniques in the Revision Fixation Setting With an Emphasis on Avoiding Varus Malalignment: A Two—Case Report

**DOI:** 10.1155/cro/9413435

**Published:** 2026-05-26

**Authors:** Aaron Jose Thomas, Ben Murphy, Conor Hurson

**Affiliations:** ^1^ School of Medicine, University College Dublin, Dublin, Ireland, ucd.ie; ^2^ Department of Trauma and Orthopaedic Surgery, St Vincent’s University Hospital, Dublin, Ireland, stvincents.ie; ^3^ Discipline of Surgery, University College Dublin, Dublin, Ireland, ucd.ie

**Keywords:** atypical femoral fractures, case report, surgical techniques, valgus alignment, varus malalignment

## Abstract

**Introduction:**

Atypical femoral fractures (AFFs) are insufficiency fractures of the lateral femoral cortex and represent a rare but significant complication of long‐term bisphosphonate or denosumab therapy. Our report explores the surgical techniques utilised to avoid varus malalignment in these two complex cases, both of which fulfilled the American Society for Bone and Mineral Research (ASBMR) criteria for AFFs.

**Case Presentation:**

Case 1 presents a woman in her early 60s with over 8 years of bisphosphonate use, who developed simultaneous AFFs. She underwent bilateral internal fixation with cephalomedullary nails. This was followed by revision surgery and a valgus correction for right femoral fracture nonunion.

Case 2 presents a man in his late 60s with prior exposure to bisphosphonates and denosumab (< 7 years), who sustained a left AFF. Initial cephalomedullary nailing was complicated by nonunion and hardware failure, necessitating revision with a valgus correction.

**Conclusion:**

Our cases emphasise the importance of recognising symptoms during follow‐up assessments and provide a detailed account of the surgical techniques employed in the revision fixation of AFFs, with a particular emphasis on achieving valgus alignment, maintaining a medial entry point and avoiding varus malalignment to optimise mechanical stability, reduce the risk of postoperative nonunion and promote fracture healing.

**Learning Points/Take Home Messages**

• Importance of surgical techniques in achieving valgus alignment and avoiding complications associated with varus malalignment in the fixation of atypical femoral fractures (AFFs).
• To recognise that fixation of AFFs is a time‐intensive procedure that demands meticulous techniques and intraoperative screening to maintain a medial entry point
• The use of a crown reamer to maintain the medial entry point and a Langenbeck retractor to prevent lateral translation to achieve satisfactory valgus alignment.

## 1. Introduction

AFFs are rare stress or insufficiency fractures typically occurring in the subtrochanteric or diaphyseal regions of the femur and are strongly associated with long‐term use of antiresorptive therapies such as bisphosphonates and denosumab [1, 2]. The presence of localised periosteal thickening of the bone cortex defined as ‘endosteal beaking’ and a ‘dreaded black line’ appearing as a transverse radiolucent line on the lateral cortex of the femur are characteristic radiographic evidence of this pathology. The former represents a stress reaction and can lead to early identification of incomplete atypical femoral fractures (iAFFs), whereas the latter is indicative of an incomplete fracture with a higher risk of progression to a complete fracture [1, 3]. Other key features of AFFs generally include an atraumatic or low‐energy trauma history, none or minimal comminution and a transverse or short oblique configuration at the fracture site (see File S1). They may initially present as either complete or incomplete fractures (iAFFs), with prodromal symptoms such as thigh or groin pain that may precede a complete fracture [2, 4].

When patients are identified to have this pathology in one of their femurs, radiographic imaging and assessment of the contralateral extremity is strongly recommended. This evaluation can be radiographic or in the form of CT scan or MRI. McKenna et al. demonstrated that iAFFs can be detected early using an extended femur length dual‐energy x‐ray absorptiometry (DEXA) with a 95% confidence interval: 1.7%–3.7% in their study [4]. Upon diagnosis of this particular injury in a patient, it is crucial that all bisphosphonates or other antiresorptive agents are discontinued.

Our case report focuses on the surgical fixation in the revision setting of AFFs and describes two patients with complete AFFs, highlighting their clinical presentation, radiographic features, with an emphasis on the surgical techniques employed to achieve valgus alignment, a medialised entry point and avoiding varus malalignment during revision fixation. This case report was prepared in accordance with the CARE reporting guidelines and the completed CARE Checklist provided.

## 2. Case 1

### 2.1. Patient Information

A woman in her early 60s with a history of osteoporosis treated with alendronate for more than 8 years. Her medical history included Crohn′s disease managed with infliximab, congenital solitary kidney, stage chronic kidney disease (CKD), hypertension, bipolar affective disorder, panic disorder with episodic paroxysmal anxiety, severe depression, gastroesophageal reflux disease (GORD) and a significant history of alcohol misuse (see File S1).

### 2.2. Clinical Findings

**Figure 1:**
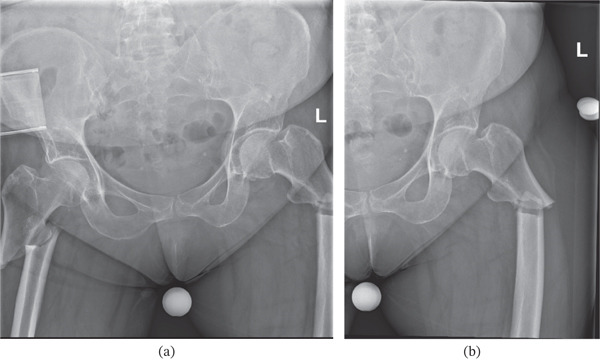
(a) Initial anteroposterior radiograph showing complete bilateral subtrochanteric femoral fractures consistent with atypical femoral fractures (AFFs) and (b) additional isolated anteroposterior radiograph of the left femur for a definitive view of bilateral fractures.

She initially presented to the emergency department following a low‐velocity mechanical fall from standing height (< 2 m) onto a concrete surface. The patient described the mechanism of injury as ‘doing the splits’ and presented with bilateral hip and inguinal pain. Initial AP radiographs revealed bilateral subtrochanteric femoral fractures (Figure 1a, 1b), consistent with AFFs associated with prolonged bisphosphonate therapy (> 8 years) and fulfilled the diagnostic criteria established by the American Society for Bone and Mineral Research (ASBMR) [2].

Each of these radiographs represents the characteristic manifestation of AFFs, displaying short oblique or transverse fractures in the subtrochanteric region with endosteal and periosteal beaking. The presence of sclerosis at the fracture lines is relatively clear, suggesting chronicity and progression of an incomplete fracture (iAFF) to a complete AFF over time (see File S1).

**Figure 2:**
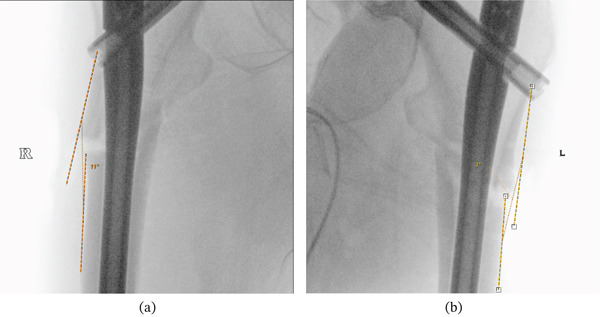
(a) Intraoperative anteroposterior screening showing initial fixation for bilateral proximal femoral fractures with long cephalomedullary nail in right femur. Noted right femur fixed in varus alignment (measured 11°) and lateral entry point and (b) intraoperative anteroposterior screening showing initial fixation for bilateral proximal femoral fractures with long cephalomedullary nail in left femur showing valgus alignment (measured < 3°) and a more medial entry point.

The patient underwent an initial open reduction and internal fixation (ORIF) with long cephalomedullary nails for bilateral proximal femoral fractures (Figure 2a,b). She remained an inpatient postoperatively for 8 weeks and subsequently attended outpatient physiotherapy. The measurements of the noted varus angle in the figures (Figure 2a,b) were made postoperatively following initial fixation to better understand the progression of the varus malalignment over time.

**Figure 3:**
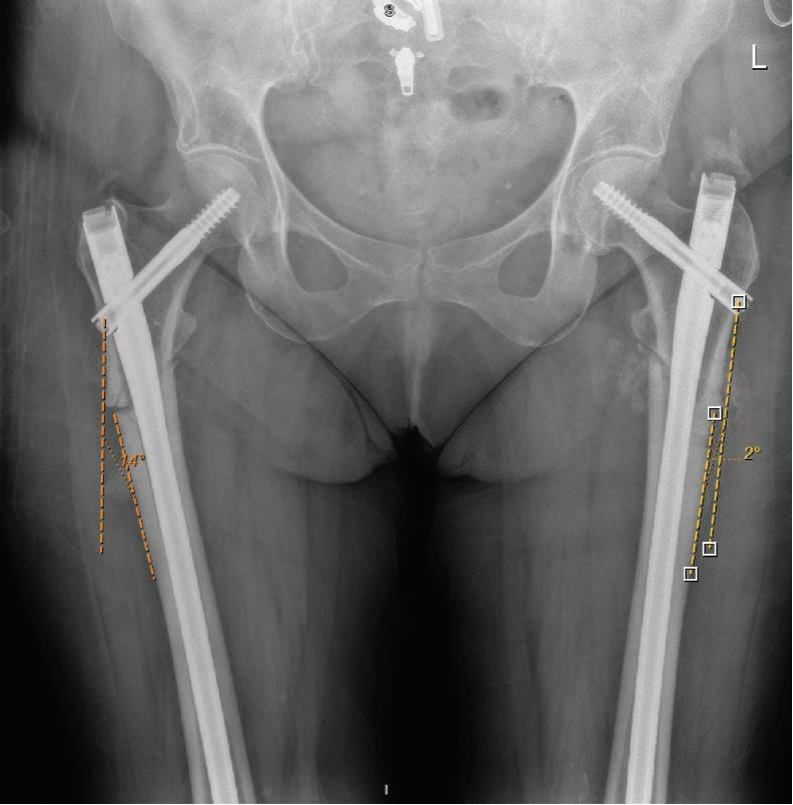
Early postoperative anteroposterior radiographic imaging showing cephalomedullary nailings for initial bilateral femoral fracture fixation with evidence of callus formation. Noted left femur fixation in valgus alignment (measured 2°) with a more medial entry point and right femur fixation in varus (measured 14°) with a lateral entry point.

During rehabilitation, she complained of right‐sided lateral thigh and anteromedial knee pain. A 1‐month postoperative radiographic imaging showed cephalomedullary nailings for initial bilateral femoral fractures with evidence of callus formation. It was noted that the right femur was fixed in varus (12°), which is a risk factor for failure when treating injuries of this pathology (Figure 3). Specifically, because these injuries take an extended period of time to heal and much longer in comparison to typical nonpathologic fractures.

**Figure 4:**
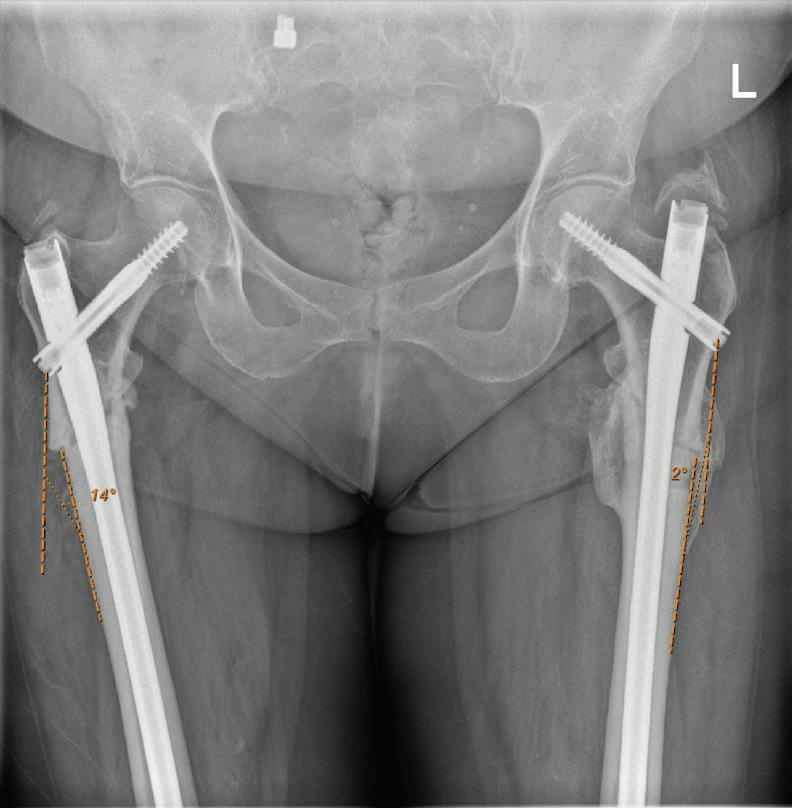
Postoperative anteroposterior radiographic imaging at 14‐month showing cephalomedullary nailings for initial bilateral femoral fracture fixation with evidence of excessive callus formation on left femur and nonunion of right femur. Noted right femur following initial fixation in varus (measured 14°).

She re‐presented to the outpatient department (OPD) 14 months after the initial procedure. She had transitioned to using a single crutch but continued to complain of persistent right‐sided lateral thigh pain, which had progressively worsened over the preceding months and a clicking sensation. Radiographic imaging showed evidence of incomplete union of the right proximal femur and excess callus formation of the left femur (Figure 4). We have noted that the excessive callus formation observed in the left femur following initial fixation reflects proper fracture healing, attributed to the more robust valgus alignment achieved during initial fixation, in contrast to the varus malalignment and subsequent nonunion observed in the right femur (see File S1). The distinctive pattern of fracture healing characterised by excessive callus formation is associated with long‐term use of bisphosphonates (> 8 years), which are potent inhibitors of bone resorption.

Revision fixation with a long cephalomedullary nail and a valgus osteotomy for correction of varus deformity was scheduled. The patient was counselled about the possibility of incomplete healing after revision, which could necessitate a proximal femoral replacement.

### 2.3. Surgical Interventions

A revision fixation of right femur was performed using long cephalomedullary nail, achieving a valgus correction of varus deformity due to a symptomatic nonunion with a surgical time of 3 h and 33 min (11:31–15:04).

**Figure 5:**
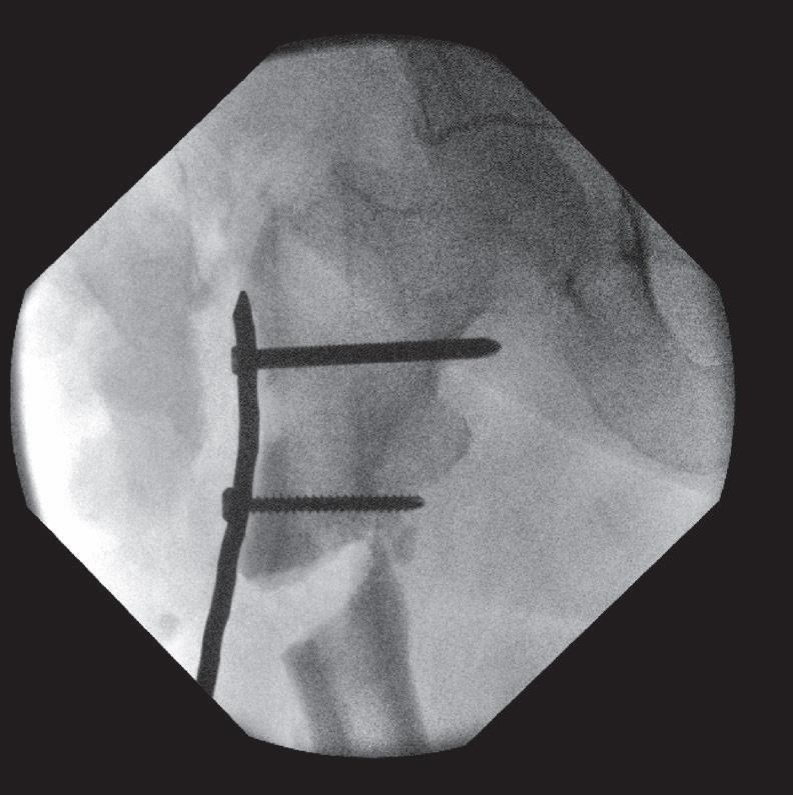
Intraoperative screening during revision fixation showing a low‐contact dynamic compression plate (LC‐DCP) that was bent to contour the greater trochanter of the proximal right femur and a locking screw that was inserted into the proximal hole to create a fixed angle construct.

The patient was positioned supine on a traction table which allowed for a controlled traction along the long axis; this decision was based on the lead surgeon′s preference and a better access to the entry point in this patient, following which the existing nail was removed using a dedicated extraction set. The varus malalignment and a mobile nonunion were noted at the fracture site. The fracture site was prepared and the edges were adequately freshened to facilitate healing. A low‐contact dynamic compression plate (LC‐DCP) was bent to contour the greater trochanter of the proximal right femur. A locking screw was inserted into the proximal hole to create a fixed angle construct (Figure 5).

**Figure 6:**
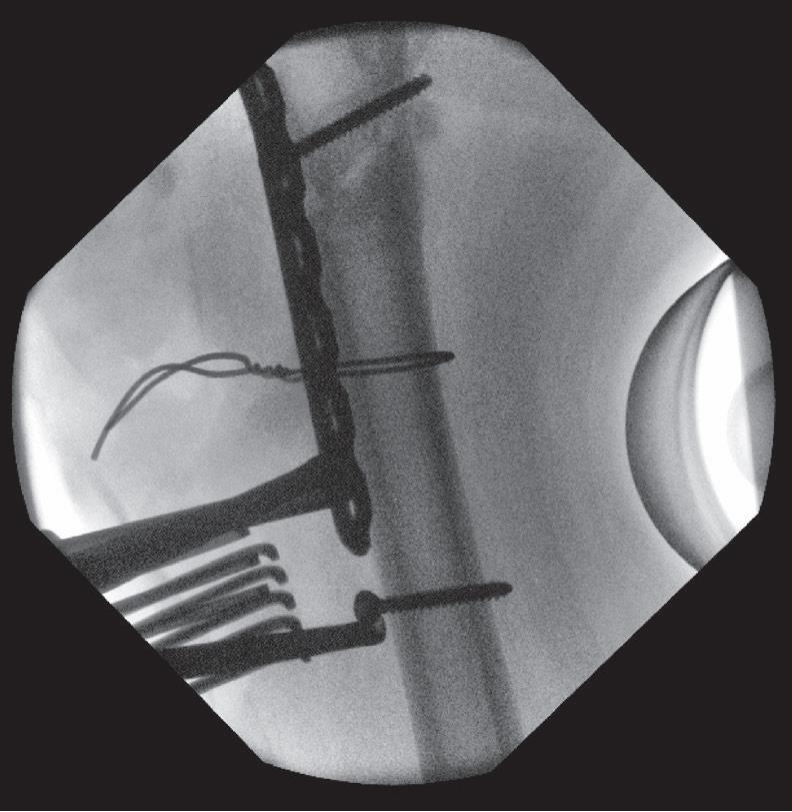
Intraoperative screening during revision fixation showing a compression clamp that was subsequently applied after reduction was maintained with a wire and distal screw insertion to achieve additional compression and valgus alignment.

The fracture was then reduced onto the distal fragment, producing a greater degree of valgus of neck shaft angle. This reduction was maintained with a wire and distal screw insertion. A compression clamp was subsequently applied to achieve additional compression and valgus alignment (Figure 6). The previously inserted intramedullary screws were then replaced with unicortical periprosthetic screws.

**Figure 7:**
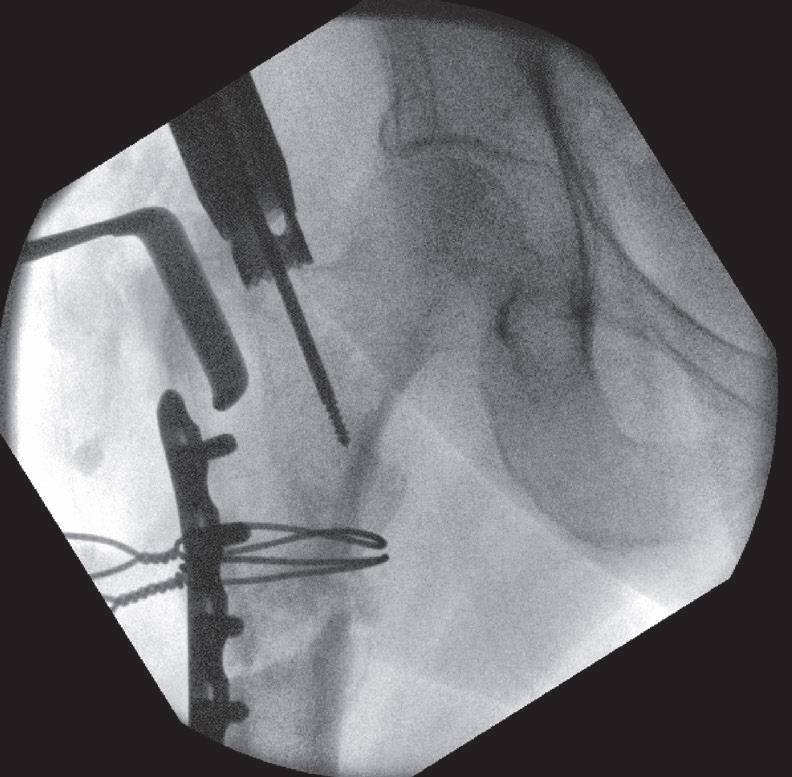
Intraoperative screening during revision fixation showing the crown reamer being used to ensure a satisfactory medial entry point, after a Langenbeck retractor was placed laterally through the previous entry site to prevent lateral translation of the reamer.

A new medial entry point was identified with the aid of a short wire, and a Langenbeck retractor was placed laterally through the previous entry site to prevent lateral translation of the reamer. A crown reamer was then utilised to ensure a satisfactory medial entry point, with attention to technique to avoid lateral deviation (Figure 7). We did not use any form of bone graft as satisfactory valgus compression and alignment had been achieved (see File S1).

**Figure 8:**
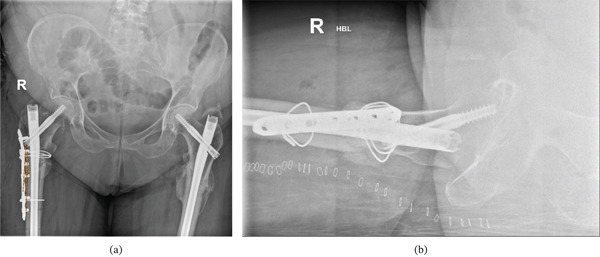
(a) Day 2 postoperative anteroposterior radiograph showing the satisfactory valgus neck‐shaft angle after revision fixation of right femur with long cephalomedullary nail and valgus correction. Noted valgus alignment postrevision fixation (measured 2°) compared with initial varus angle measured 14°(Figure 3) and (b) Day 2 postoperative lateral radiograph of right femur following revision fixation with long cephalomedullary nail and valgus correction.

After the cephalomedullary nail was inserted, the plate was left in situ with screws and wires securely positioned. A portable negative pressure wound therapy (NPWT) device (PICO dressing) was applied. Radiographic imaging performed on Day 2 postoperatively showed a satisfactory valgus neck‐shaft angle (Figure 8a,b).

### 2.4. Biochemistry

On the most recent admission, biochemistry and bone profile investigations revealed haemoglobin 13.5 g/dL (12–15.5 g/dL), white cell count 13.2 × 10^9^/L (4–11 × 10^9^/L), platelets 731 × 10^9^/L (150–400 × 10^9^/L), urea 7.6 mmol/L (2.5–7.8 mmol/L), sodium 134 mmol/L (135–145 mmol/L), potassium 4.2 mmol/L (3.5–5.0 mmol/L), creatinine 132 *μ*mol/L (45–90 *μ*mol/L), creatine kinase 852 IU/L (25‐200), CRP 31.2 mg/L, HbA1c 39 mmol/mol (20–42) and eGFR 38 mL/min/1.73 m^2^ indicative of Stage 3b CKD.

Serum calcium measured 2.20 mmol/L (2.2–2.6 mmol/L), iron 8.3 *μ*mol/L (5.8–34.5), magnesium 1.05 mmol/L (0.70–1.00), ferritin 218 (13–150), chloride 97 mmol/L (95–108), ALP 86 IU/L (35–104 U/L), ALT 72 IU/L (8–41), GGT 256 IU/L (6–42), CRP 108.4 mg/L (< 5 mg/L), TSH 2.10 mIU/L (0.4–4.0 mIU/L) and 25(OH)Vitamin D 85 nmol/L (30–125 nmol/L) showing adequate Vitamin D levels following vitamin D repletion for previously identified deficiency, under review by the endocrinology team.

A recent DEXA scan was indicative of osteopenia and showed the following *T*‐scores: lumbar spine −2.0, left femoral neck −2.1 and left total hip −0.9, consistent with a diagnosis of osteopenia. There has been an interval decrease in bone mineral density (BMD) of lumbar spine from 0.934 to 0.826, an 11.5% change and an interval decrease in left total hip BMD from 0.875 to 0.87, a 5.5% change compared with previous years.

## 3. Case 2

### 3.1. Patient Information

A man in his late 60s with osteoporosis, previously treated with alendronate (treatment dates unclear) and maintained on denosumab for 6 years. His medical history included skeleto‐facial dysplasia, severe kyphoscoliosis, and mild lower limb spasticity related to complications from premature birth. He was also under ENT follow‐up for unilateral left‐sided hearing loss. Psychiatric history includes low mood and intermittent mild delusional perception, currently managed with escitalopram. A family history reveals autoimmune arthropathy in four of eight siblings, three of whom are currently receiving biologic therapy; however, the patient tested negative for autoimmune markers (see File S1).

### 3.2. Clinical Findings

**Figure 9:**
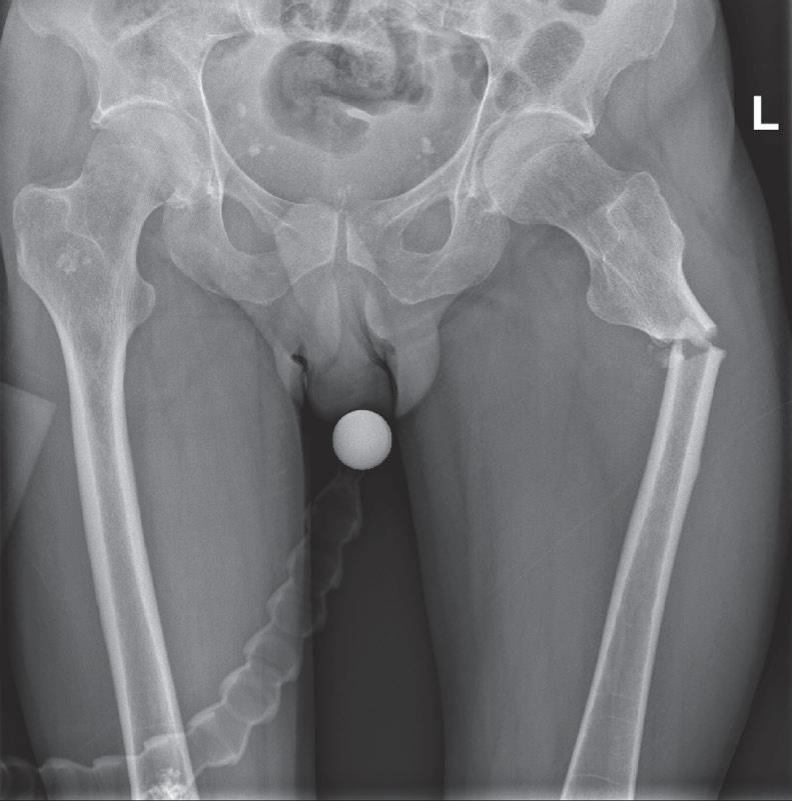
Initial anteroposterior radiograph following presentation to the emergency department showing a complete lateral cortex transverse fracture of the left femur.

He initially presented to the emergency department with atraumatic thigh‐to‐groin pain and inability to bear weight on the left leg. Initial management included analgesia, followed by radiographic imaging that showed a complete lateral cortex transverse fracture of the left femur (Figure 9). The findings of the initial radiograph were consistent with a left atypical subtrochanteric femoral fracture, fulfilling ASBMR diagnostic criteria [2].

**Figure 10:**
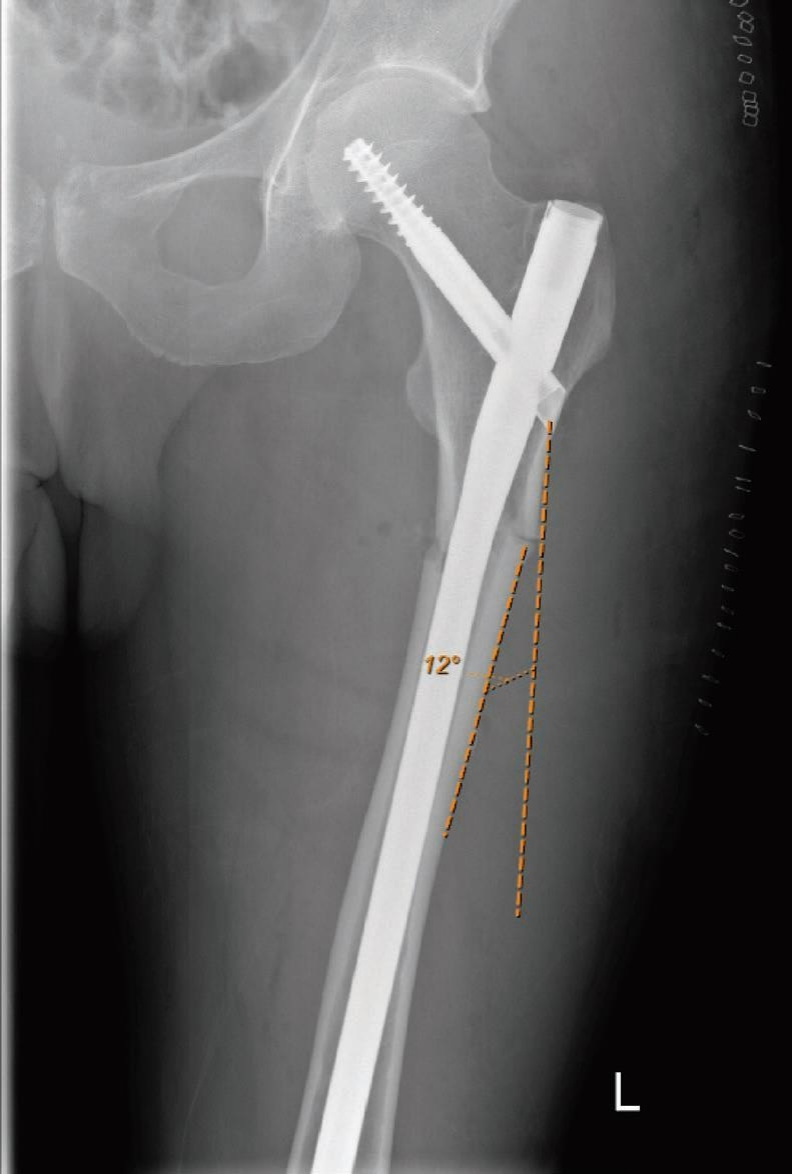
Immediate postoperative anteroposterior imaging following cephalomedullary nailing for left atypical subtrochanteric femoral fracture. Noted varus fixation of left femur (measured 12°) and a lateral entry point.

He was admitted and promptly underwent an initial surgical fixation with left cephalomedullary nail insertion; postoperative imaging noted that the left femur was fixed in varus (12°), similar to Case 1 (Figure 10). Denosumab was discontinued postoperatively, and a drug holiday was initiated.

**Figure 11:**
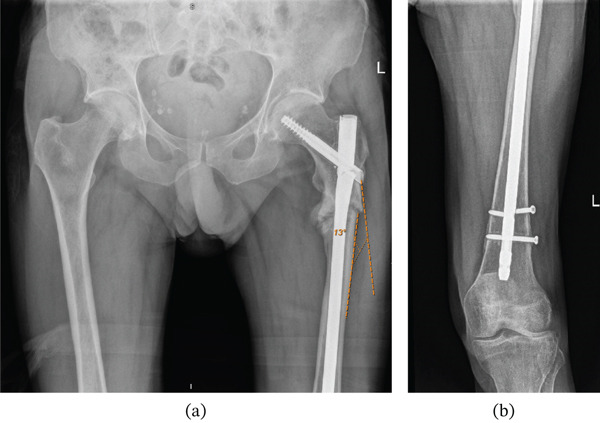
(a) 8‐month postoperative anteroposterior radiographic imaging after initial procedure showing nonunion at the left femur fracture site and a broken proximal nail. Noted varus deformity (measured 13°) and (b) 8‐month postoperative anteroposterior radiographic imaging after initial procedure showing a broken distal locking screw.

After an initial 8‐month pain‐free period, the patient re‐presented to the OPD with progressive left hip pain and an antalgic gait (see File S1). Radiographic imaging (x‐ray and CT) showed residual varus deformity following initial fixation (measured angle 15.24°), nonunion at the fracture site and mechanical failure of the proximal nail (Figure 11a) and a broken distal locking screw (Figure 11b). Endocrine and biochemical investigations in the initial presentation revealed persistently suppressed serum C‐terminal telopeptide (CTX) levels, indicative of low bone turnover. Revision intramedullary nailing with valgus correction to bring the proximal varus fragment into valgus alignment was scheduled.

### 3.3. Surgical Interventions

We performed a revision cephalomedullary nailing of the left femur with a valgus correction for nonunion of the atypical femoral fracture. The total surgical time was 4 h and 33 min (13:25–17:58).

In this patient, preoperative imaging (Figure 11) demonstrated that there was a broken proximal trochanteric femoral nail (TFN), a broken distal locking screw and a varus deformity (measured 13°). The patient was positioned in the lateral decubitus position, and a traction table was not used in this case due to surgeon preference and an easier entry point access. The fractured nail was only removed after a LCDCP had been contoured and positioned, with a proximal locking screw on the anterolateral aspect of the femoral head and neck to avoid future interference with the cephalomedullary nail. Subsequently, the broken proximal TFN nail and broken distal locking screw were removed. The fracture site was then prepared, and a more valgus neck‐shaft angle was achieved by reducing the plate to the distal femoral shaft using wires and screws. A compression clamp was used to apply additional compression and achieve a neck shaft angle in a greater degree of valgus.

**Figure 12:**
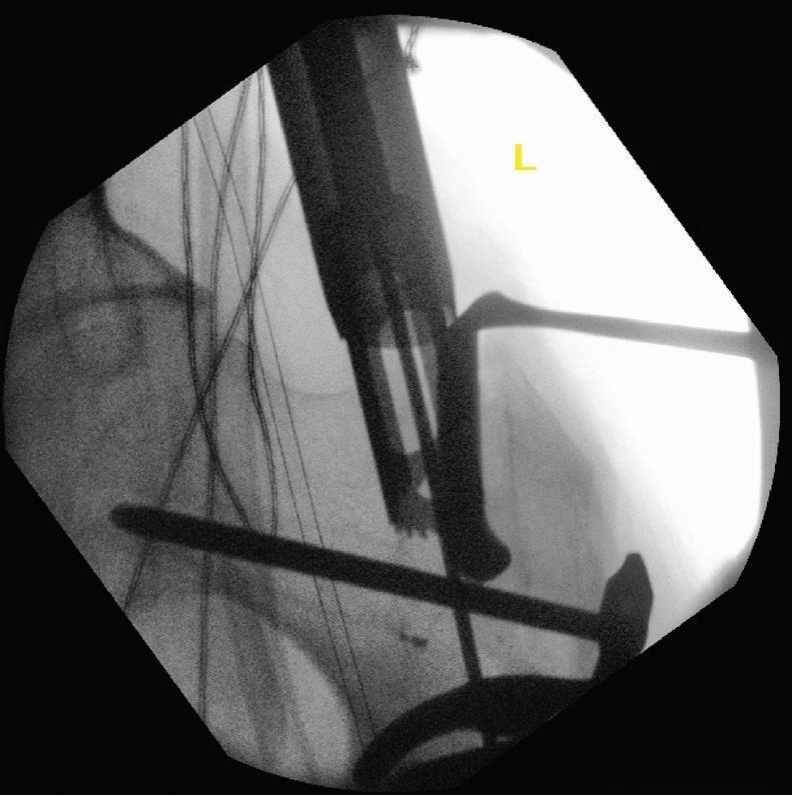
Intraoperative screening during revision fixation showing the use of a crown reamer for optimal medial entry trajectory and a Langenbeck retractor on the lateral aspect of the entry hole to avoid lateral translation of the reamer. It shows the confluence of metalwork observed on advancing the guidewire through the new medialised entry point.

After achieving satisfactory valgus alignment, a new medialised entry point was identified. A crown reamer was used to ensure optimal medial entry trajectory was maintained and a Langenbeck retractor was then placed on the lateral aspect of the entry hole to avoid lateral translation of the reamer. However, unlike in Case 1, resistance was initially encountered during advancement of the guidewire while accessing the newly medialized entry point (see File S1). Repetitive intraoperative screening revealed that the screws used to secure the contoured LC‐DCP were obstructing the new medial entry point and were the source of the resistance encountered on advancing the guidewire (Figure 12). These screws were subsequently removed due to confluence of metalwork, whereas valgus compression was maintained using cerclage wires.

**Figure 13:**
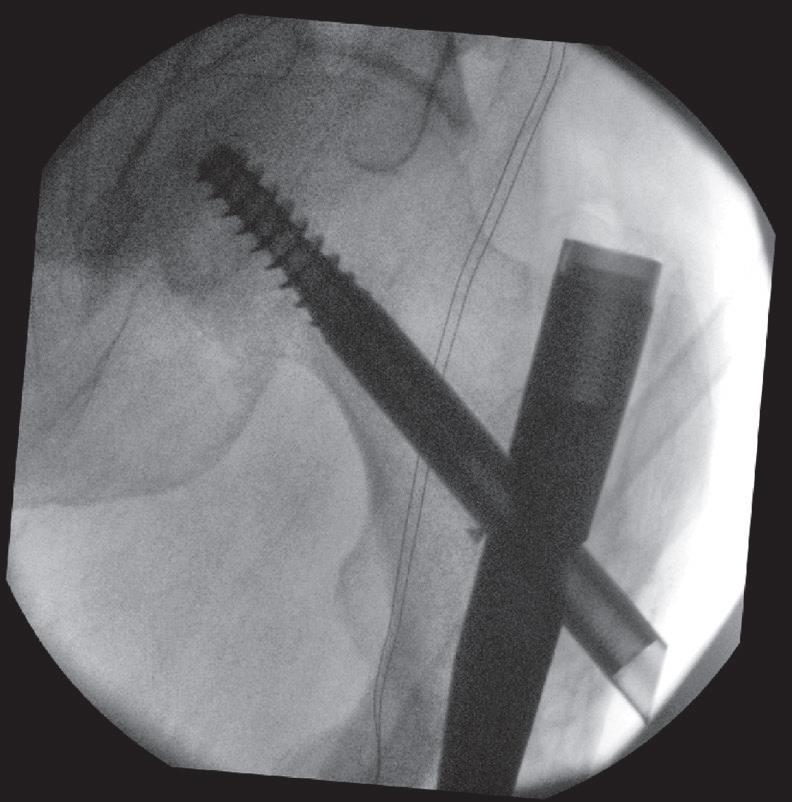
Intraoperative screening during revision fixation showing the medialised entry point and cephalomedullary nailing in the left femur with satisfactory valgus alignment.

**Figure 14:**
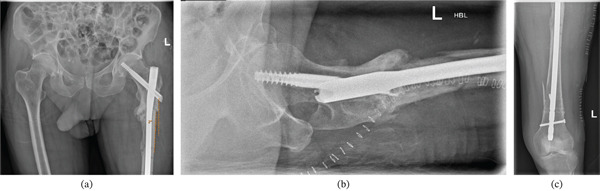
(a) Early postoperative anteroposterior radiograph showing cephalomedullary nail insertion and the satisfactory valgus alignment (measured 2°) in comparison to preoperative (Figure 11) radiographic imaging (measured angle 13°), (b) early postoperative lateral radiograph of left femur following revision fixation with cephalomedullary nail insertion and valgus correction. (c) early postoperative anteroposterior radiograph of left femur following revision fixation with cephalomedullary nail insertion, valgus correction and distal locking screw.

A long cephalomedullary nail was inserted with a distal screw placed in the dynamic hole; repeat intraoperative screening demonstrated a medialised entry point and a satisfactory valgus alignment (Figure 13). The contoured LC‐DCP held by cerclage wires was then removed as satisfactory valgus compression was achieved. Postoperative radiographic imaging showed a satisfactory valgus alignment (measured angle 2°) in comparison to preoperative imaging (measured angle 13°) and distal locking screw (Figure 14a, c).

### 3.4. Biochemistry

On the most recent admission, biochemistry and bone profile investigations revealed haemoglobin 10.2 g/dL (12–15.5 g/dL), white cell count 13.3 × 10^9^/L (4–11 × 10^9^/L), platelets 154 × 10^9^/L (150–400 × 10^9^/L), urea 6.2 mmol/L (2.5–7.8 mmol/L), sodium 144 mmol/L (135–145 mmol/L), eGFR > 90 mL/min/1.73 m^2^, creatine (enzymatic) 65 *μ*mol/L (59–104), and HbA1c 36 mmol/mol (20–42).

Serum calcium measured 2.41 mmol/L (2.2–2.6 mmol/L), magnesium 0.83 mmol/L (0.70–1.00), phosphate 1.05 mmol/L (0.8–1.50), chloride 101 mmol/L (95–108), ALP 77 IU/L (35–104 U/L), ALT 17 IU/L (8–41), GGT 19 IU/L (6–42), CRP 84.6 mg/L (< 5 mg/L), albumin 37 g/L (35–50), total cholesterol/HDL ratio 2.25 and total PSA 2.97 (0–3.99).

The recent DEXA scan demonstrated T‐scores of −2.5 in the lumbar spine, −2.6 in the left femoral neck and −1.7 in the left total hip, indicative of osteoporosis. Notably, there was significant improvement in BMD of 5.1% in the lumbar spine and 6.5% in the left hip since the previous scan performed 4 years prior. Most recent C‐terminal telopeptide CTX‐1 levels showed an increase to 0.299 *μ*g/L (0.015–0.584) compared with 0.097 *μ*g/L the previous year.

## 4. Outcome and Follow‐Up

Postoperative radiographs in both Cases 1 and 2 demonstrated satisfactory valgus alignment. Physiotherapy was subsequently resumed with the objective of facilitating early weight‐bearing, using appropriate mobility aids—either a walking frame or two crutches, as individually prescribed. It is noteworthy that, in Case 1, the patient reported a marked improvement in the previously described right‐sided lateral thigh pain during the early postoperative rehabilitative period following revision fixation. Patients were scheduled for clip removal by their general practitioners within 2 weeks of discharge, followed by consultations at one and 6 months in the OPD for complete assessment of bone turnover markers, complemented by serial imaging to monitor for signs of contralateral hip involvement or delayed union. The evaluation of fracture union and functional recovery remains under active surveillance and follow‐up (see File S1).

## 5. Discussion

Our cases highlight the critical surgical techniques in the revision fixation of bisphosphonate‐associated AFFs. The importance of achieving valgus alignment while meticulously avoiding varus malalignment is crucial and should begin with an initial anatomic fracture reduction, facilitated by the use of clamps and cables, or augmented with plates, as appropriate. Accurate placement of the entry guide wire plays a pivotal role in avoiding varus malalignment. Varus malalignment, a significant complication in the fixation of AFFs, increases the risk of nonunion at the fracture site and predisposes to secondary mechanical failure [5]. The use of intraoperative screening to accurately establish a medial entry point using a crown reamer and a Langenbeck retractor to prevent lateral translation was a critical surgical technique employed in the management of AFFs in our cases. The fixation of AFFs can be time‐intensive procedures, as reflected by the surgical durations of 3 h and 33 min and 4 h and 33 min for Cases 1 and 2, respectively.

We show through these cases that the initial fixation in varus malalignment and a lateral entry point had predictably failed, then revised with valgus alignment and a medial entry point for a more robust fixation and improved postoperative outcomes for the patient [5, 6]. This highlights the importance of obtaining an appropriate anatomic or valgus reduction during the initial procedure. It has been shown that adequate reaming of the endosteal beaking, notably in the proximal femur, during nail entry facilitates a medialised entry point and allows the intramedullary device to be placed directly along the lateral cortex, promoting appropriate anatomic or valgus alignment. This can be achieved using a reamer or through an open technique with a burr and curette [7].

Femoral geometry is recognised as an important factor in the development of AFFs, with whole‐femur bowing, a known risk factor for atypical femoral features, being associated with increased lateral cortical stress and posing challenges in achieving and maintaining valgus alignment [8]. Whole‐femur bowing was not quantitatively assessed, this was limited by the retrospective nature of the initial presentation of the cases and bilateral femoral involvement in Case 1, these factors restricted standardised measurement. This represents a limitation in our report, as it prevents the contribution of femoral geometry to fracture occurrence and mechanical failure from being determined in these cases.

The profound reduction in bone remodelling caused by long‐term use of bisphosphonates is a key biological mechanism in the development of AFFs. Although there is an increase of bone mass in the cancellous region that reduces the risk of osteoporotic fractures with bisphosphonate use, the cortical compartment faces an increased risk of fragility fractures, hypothetically predisposing to AFFs [9]. These factors show that the management of AFFs is multifactorial and involves both mechanical and biological considerations, including bone quality, remodelling activity and metabolic status. In our report, comprehensive biological assessment was limited. Although BMD and biochemistry data were available, the absence of complete biochemical and bone turnover markers for both cases limits interpretation of the biological contribution to fracture development, delayed healing, and fixation failure. This represents a limitation in our cases and highlights the importance of integrating both biological and mechanical factors when managing AFFs.

Pharmacological agents such as teriparatide may accelerate fracture healing in AFFs [10]. In Case 1, current guidelines indicate caution and close monitoring of calcium and parathyroid hormone (PTH) levels in moderate kidney disease [11], taking into account the patient′s history and ongoing management of Stage 3b CKD. In Case 2, teriparatide monotherapy is not recommended after denosumab withdrawal, as this transition is associated with extensive skeletal remodelling and even greater bone loss than that observed when denosumab is discontinued without a drug transition [12]. However, both patients are currently under multidisciplinary review and ongoing surveillance to assess the suitability of teriparatide therapy to facilitate fracture healing.

Our report focuses on the surgical techniques in revision fixation and mechanical factors contributing to fixation failure. However, these findings should be interpreted within the broader context that encapsulates biological and surgical management of AFFs. This highlights that optimal management of AFFs extends beyond surgical technique to maintain valgus alignment and further requires a patient‐centred, multidisciplinary approach involving endocrinology review and serial monitoring of biological activity to deliver optimal patient care. This discussion was prepared in accordance with the CARE reporting guidelines (see File S1).

## 6. Conclusion

In conclusion, our cases highlight the surgical techniques utilised in the revision fixation of AFFs, including the use of a crown reamer to maintain a medialised entry point and adjunctive devices to achieve satisfactory valgus alignment.

They also emphasise that avoiding varus malalignment in the initial fixation can decrease the risk of nonunion at fracture site and secondary mechanical failure, optimising patient outcomes, fracture healing, enhancing postoperative recovery and reducing postoperative complications.

## Funding

No funding was received for this manuscript.

## Ethics Statement

A formal ethical approval was not required for this case report. Written informed consent and waivers were obtained from the patients for publication of clinical details and accompanying images.

## Consent

The authors obtained informed consent from both individual patients.

## Conflicts of Interest

The authors declare no conflicts of interest.

## Supporting information


**Supporting Information.** Additional supporting information can be found online in the Supporting Information section. File S1: A completed CARE Checklist is provided as supporting information to improve accuracy, to support methodological transparency and compliance of this case report with the CARE Case Report Guidelines [13].

## Data Availability

The data that support the findings of this study are available from the corresponding author upon reasonable request.
