# Supplementary material for: Bisphosphonate‐Associated Atypical Femoral Fractures: A Description of Surgical Techniques in the Revision Fixation Setting With an Emphasis on Avoiding Varus Malalignment: A Two—Case Report
Source: Case Rep Orthop. 2026 May 26;2026:9413435. doi: 10.1155/cro/9413435 (PMC13213072; doi:10.1155/cro/9413435)
Supplement: Supplementary file 1 — Supporting Information. Additional supporting information can be found online in the Supporting Information section. File S1: A completed CARE Checklist is provided as supporting information to improve accuracy, to support methodological transparency and compliance of this case report with the CARE Case Report Guidelines [13]. [file CRO-2026-9413435-s001.pdf]

CARE case report guidelines

CARE Checklist of information to include when writing a case report

(cc) BY-NC-ND

Topic	Item	Checklist item description	Reported on Line
Title	1	The diagnosis or intervention of primary focus followed by the words "case report"	1/2
Key Words	2	2 to 5 key words that identify diagnoses or interventions in this case report, including "case report"	34/35
Abstract (no references)	3a	Introduction: What is unique about this case and what does it add to the scientific literature?	14-18
	3b	Main symptoms and/or important clinical findings	21/25
	3c	The main diagnoses, therapeutic interventions, and outcomes	22/26
	3d	Conclusion—What is the main "take-away" lesson(s) from this case?	28-32
Introduction	4	One or two paragraphs summarizing why this case is unique (**may include references**)	46-72
Patient Information	5a	De-identified patient specific information.	75-76 / 238-239
	5b	Primary concerns and symptoms of the patient.	83-85 / 247-251
	5c	Medical, family, and psycho-social history including relevant genetic information	76-79 / 239-245
	5d	Relevant past interventions with outcomes	105-110 / 257-260
Clinical Findings	6	Describe significant physical examination (PE) and important clinical findings.	136-139 / 267-271
Timeline	7	Historical and current information from this episode of care organized as a timeline	135 / 267
Diagnostic Assessment	8a	Diagnostic testing (such as PE, laboratory testing, imaging, surveys).	215-233 / 344-360
	8b	Diagnostic challenges (such as access to testing, financial, or cultural)	406-408
	8c	Diagnosis (including other diagnoses considered)	138-139 / 268-271
	8d	Prognosis (such as staging in oncology) where applicable	-
Therapeutic Intervention	9a	Types of therapeutic intervention (such as pharmacologic, surgical, preventive, self-care)	158-159 / 287-288
	9b	Administration of therapeutic intervention (such as dosage, strength, duration)	160 / 289
	9c	Changes in therapeutic intervention (with rationale)	293-294 / 311-313
Follow-up and Outcomes	10a	Clinician and patient-assessed outcomes (if available)	366-376
	10b	Important follow-up diagnostic and other test results	366-376
	10c	Intervention adherence and tolerability (How was this assessed?)	375-376
	10d	Adverse and unanticipated events	-
Discussion	11a	A scientific discussion of the strengths AND limitations associated with this case report	409-410/ 418-424
	11b	Discussion of the relevant medical literature **with references**.	380-434
	11c	The scientific rationale for any conclusions (including assessment of possible causes)	403-410/ 412-418/ 426-432
	11d	The primary "take-away" lessons of this case report (without references) in a one paragraph conclusion	436-442
Patient Perspective	12	The patient should share their perspective in one to two paragraphs on the treatment(s) they received	369-371
Informed Consent	13	Did the patient give informed consent? Please provide if requested	Yes ☒ No ☐
